# Functional Role of VCAM-1 Targeted Flavonoid-Loaded Lipid Nanoemulsions in Reducing Endothelium Inflammation

**DOI:** 10.3390/pharmaceutics11080391

**Published:** 2019-08-03

**Authors:** Elena Valeria Fuior, Mariana Deleanu, Cristina Ana Constantinescu, Daniela Rebleanu, Geanina Voicu, Maya Simionescu, Manuela Calin

**Affiliations:** 1 Institute of Cellular Biology and Pathology “Nicolae Simionescu”, 050568 Bucharest, Romania; 2 Faculty of Biotechnologies, University of Agronomic Sciences and Veterinary Medicine (UASVM), 011464 Bucharest, Romania; 3 Faculty of Veterinary Medicine, University of Agronomic Sciences and Veterinary Medicine (UASVM), 050097 Bucharest, Romania

**Keywords:** cell-targeting peptide, naringenin, hesperetin, endothelium, lipid nanoemulsions, nanoparticle

## Abstract

Citrus flavonoids have well-documented protective effects on cardiovascular system, but the poor water solubility and reduced bioavailability restrict their therapeutic use. We aimed to overcome these limitations and encapsulated naringenin and hesperetin into lipid nanoemulsions (LNs), targeted to vascular cell adhesion molecule-1 (VCAM-1), which is expressed on activated endothelial cells (ECs). LNs were characterized by a hydrodynamic size of ~200 nm, negative zeta potential, an encapsulation efficiency of flavonoids higher than 80%, good in vitro stability and steady release of the cargo. The LNs were neither cytotoxic to human ECs line EA.hy926, nor provoked in vitro lysis of murine erithrocytes. Then, we tested whether these nanoformulations reduce tumor necrosis factor-alpha (TNF-α) induced EC-activation. We found that flavonoid-loaded LNs, either non-targeted or targeted to the endothelium, were taken up by the EA.hy926 cells in a dose-dependent manner, but dependent on TNF-α only in the case of endothelium-targeted LNs. Moreover, these nanoparticles inhibited both the adhesion and transmigration of THP-1 monocytes on/through activated ECs, by mechanisms involving a reduced expression of the pro-inflammatory chemokine monocyte chemotactic protein 1 (MCP-1) and diminished nuclear translocation of nuclear factor kappa-light-chain-enhancer of activated B cells (NF-κB).

## 1. Introduction

Naringenin and hesperetin, the main citrus flavonoids, either in their free form or as the corresponding naturally occurring glycosides (Table 1), are intensely investigated recently for their therapeutic potential in atherosclerosis and type II diabetes, two of the dreadful pandemics of the developed society. Citrus flavonoids exert a large spectrum of activities such as anti-oxidant, anti-inflammatory, glucose- and lipid-lowering and improve the metabolic parameters: they correct dyslipidemia, increase glucose tolerance and insulin sensitivity, diminish the inflammation and the accumulation of adipose tissue [1,2].

A series of in vitro and in vivo studies indicated that adhesion of monocytes to endothelial cells, an early pro-inflammatory step in atherogenesis, is reduced by the treatment with citrus flavanones via the decreased expression of cell adhesion molecules (i.e., vascular cell adhesion molecule-1 (VCAM-1), intercellular Adhesion Molecule 1 (ICAM-1) and E-selectin) and pro-inflammatory cytokines (such as fractalkine also known as chemokine (C-X3-C motif) ligand 1 (CX3CL1), monocyte chemotactic protein 1 (MCP-1) and regulated on activation, normal T cell expressed and secreted (RANTES) [3,4,5], mediated at least partially through the diminished nuclear translocation of nuclear factor kappa-light-chain-enhancer of activated B cells (NF-κB) transcription factor [6].

The drawbacks that reduce the efficacy of oral administration of citrus flavanones are poor water solubility (0.214 and 0.273 g/L for naringenin and, respectively, hesperetin as provided by [7], but even lower according to other sources) and limited oral bioavailability [8,9]. The glycosidic forms of citrus flavonoids are converted by the intestinal microbiota into the free aglycones, which are taken up by the enterocytes, but there are large interindividual variations in the deglycosylation capacity [10] which may result in different profiles of bioavailable flavonoids.

These limitations call upon alternative formulations and/or delivery routes to improve the pharmacokinetics of these compounds. One successful strategy was the complexation of naringenin with β-cyclodextrin, which augmented its solubility 400-fold, a circumstance that subsequently brought about a ~15-fold increase in flavonoid plasma concentration and improved hypolipemic and hypoglycemic effects as compared to the free substance orally administered to Sprague-Dawley rats [11]. Oral administration of naringenin-loaded liposomes to Kunming mice increased ~14-fold naringenin bioavailability, with a predominant hepatic distribution [12]. Naringenin incorporated into chitosan core-shell nanoparticles coated with alginate proved non-toxic and efficiently reduced hyperglycemia in streptozotocin-induced diabetic rats upon oral administration [13].

Prompted by the beneficial anti-inflammatory effects described for naringenin and hesperetin, we envisaged that incorporation in a nanocarrier, specifically directed towards inflamed sites of the vasculature, would increase their efficacy after intravenous delivery. Currently, there are no data regarding the intravenous administration of naringenin and hesperetin incorporated in nanosystems. Nevertheless, based on our previous data [14], we foresee that the formulation of polyphenols into targeted nanocarriers would significantly increase their concentration to sites susceptible to vascular inflammation.

Cell adhesion molecules are attractive targets, since their expression is inducible on the surface of activated endothelial cells in pathologically altered vasculature [15,16]. Various antibody-based or peptide-based theranostic nanocarriers targeting VCAM-1, ICAM-1, E- and P-selectin, or platelet endothelial cell adhesion molecule (PECAM-1) are described in the literature to deliver their cargo to endothelial cells in in vitro and in vivo models of atherosclerosis [17,18,19,20].

We had previously used a peptide with affinity for VCAM-1 to successfully target liposomes to activated endothelium in atherosclerosis [21] and cancer [22].

Taking advantage of these previous data, we hypothesized that the encapsulation of the two citrus flavanones, naringenin and hesperetin, into lipid nanoemulsions directed towards VCAM-1 could be employed successfully for the reduction of tumor necrosis factor-alpha (TNF-α)—induced endothelial cell inflammation. We report herein that PEGylated LNs coupled with a VCAM-1 recognizing peptide and encapsulating naringenin (V-Nar/LNs) or hesperetin (V-Hesp/LNs) were taken up by activated ECs in a dose-, time-, and TNF-α dependent manner. V-Nar/LNs and V-Hesp/LNs exerted a significantly higher percentage of inhibition of monocyte adhesion and transmigration to/through the TNF-α—activated endothelium as compared with non-targeted and free flavanones, used at the same concentration. To the best of our knowledge, this is the first study showing that VCAM-1 targeted lipid nanoemulsions can deliver polyphenols to activated ECs and have the functional capacity to reduce monocyte infiltration through activated ECs by reducing the nuclear translocation of NF-kB and the production of MCP-1 chemokine.

## 2. Materials and Methods

### 2.1. Reagents

The commercial sources of the main reagents and consumables used in this study were as follows: naringenin (purity > 95%), hesperetin (purity > 95%), soybean oil, Dulbecco’s modified Eagle’s medium (DMEM) and RPMI medium were from SIGMA-Aldrich (St. Louis, MO, USA).; soy phosphatidyl choline (SPC), 1,2-dipalmitoyl-sn-glycero-3-phosphoethanolamine-*N*-(lissamine rhodamine B sulfonyl) (ammonium salt) (Rhodamine-PE) and1,2-distearoyl-sn-glycero-3-phosphoethanolamine-*N*-[Maleimide(PolyethyleneGlycol)2000] (Ammonium salt) (Mal-PEG-DSPE) from Avanti Polar Lipids (Alabaster, AL, USA); recombinant human tumor necrosis factor-alpha (TNF-α) from R&D Systems, Inc. (Abingdon, UK); VCAM-1 recognition peptide of sequence H_2_N-VHPKQHRGGSKGCC–COOH from GeneCust (Dudelange, Luxembourg); glycerin from Carl Roth (GmBH, Germany); 2,7-bis(2-carboxyethyl)-5(6)-carboxyfluorescein acetoxymethyl ester (BCECF-AM); 2,3-Bis-(2-Methoxy-4-Nitro-5-Sulfophenyl)-2*H*-Tetrazolium-5-Carboxanilide (XTT) and Tris (2-carboxyethyl) phosphine (TCEP) from ThermoFisher Scientific (Rockford, IL, USA), fetal bovine serum (FBS), penicillin and streptomycin from Gibco (ThermoFisher Scientific); SpectraPor dialysis membrane (cut-off 100–500 Da) was from Spectrum Labs (Spectrum Europe BV, Breda, The Netherlands), 100 kDa cut-off Amicon centrifugal filter columns and transmigration cell culture inserts from Millipore (Billerica, MA, USA); cell culture dishes were from TPP^®^ (Trasadingen, Switzerland). Deionized water (18.2 MΏ/cm) was obtained in house using Milli-Q system from Millipore (Watford, UK). HPLC grade solvents were from Merck (Kenilworth, NJ, USA).

### 2.2. Preparation of Non-Targeted Flavonoid-Loaded Nanoemulsions

A previously optimized method for the preparation of nanoemulsions was employed [14]. The nanoemulsions formula contains the organic phase consisting of SPC (9.8 mM), Mal-PEG-DSPE (0.2 mM) and 0.5% *v*/*v* soybean oil, reconstituted in an aqueous phase containing glycerin, as surfactant. Lipid nanoemulsions were prepared by the ultrasonication method. Briefly, the protocol was carried out as follows: the organic phase, and a fixed volume of ethanol (60 µL) containing or not the flavonoid, was evaporated on a rotary evaporator (Laborota 4000, Heidolph) at 40 °C, in vacuum. The residual was resuspended in the aqueous phase, containing 1 mL of water and 10% glycerin. The coarse emulsion was sonicated for 10 min using a UPH200H probe-type sonicator from Hielscher. The nanoemulsions was further centrifuged using a 100 kDa cut-off Amicon centrifugal column to remove non-encapsulated flavonoid and traces of organic solvents. Light exposure was minimized to prevent the photodegradation of flavonoids. The lipid nanoemulsions were characterized by determining the size and zeta potential using a ZetaSizer NanoZS instrument (Malvern Instruments, Malvern, UK).

To fluorescently label the lipid nanoemulsions, 1.5% (mole/mole) Rhodamine-PE was added as an ethanol solution subsequent to LN preparation and incubated 30 min at room temperature in the dark.

### 2.3. Preparation of VCAM-1 Targeted Flavonoid-Loaded Nanoemulsions

The VCAM-1 recognition peptide, described by Kelly et al. [23], was coupled to the flavonoid-loaded lipid nanoemulsions as previously described [21]. Before coupling, to ensure that the sulfhydryl groups of the peptide are available in the reduced form, the peptide was treated with a 25-fold molar excess TCEP for 2 h at room temperature, followed by overnight dialysis (membrane cut-off 100–500 Da) at 4 °C against coupling buffer (20 mM sodium phosphate, pH 6.7, 30 mM NaCl and 2 mM EDTA). The coupling of peptide thiols to the maleimide group at the distal end of Mal-PEG-DSPE on the nanoparticle’s surface was achieved by overnight incubation at 4 °C. Non-reacted maleimide groups were blocked by the addition of a 100-fold molar excess of L-cysteine for 30 min at room temperature, followed by the removal of the non-reacted cysteine by employing 100 kDa cut-off Amicon centrifugal filters.

### 2.4. Characterization of Flavonoid-Loaded Lipid Nanoemulsions

#### 2.4.1. Size and Zeta Potential

The size of lipid nanoemulsions was determined by dynamic light scattering (DLS) on a Zetasizer Nano ZS instrument (ZEN 3600) (Malvern Instruments, Malvern, UK) equipped with a 633 nm laser. Measurements were performed under automatic attenuator selection at a 10 µM total lipid concentration in water at 25 °C, with the following physico-chemical parameters utilized: viscosity of dispersant (water) 0.8872 cP, dispersant dielectric constant 78.5, refractive indices: 1.45 for phospholipids and 1.33 for dispersant. One individual record represents the average of 15 measurements per sample and for each sample three such records were acquired. The reported Z-averages of intensity distributions (in nm) represent averages of these measurements, together with the PDI (polydispersity index), which reflects the homogeneity of the sample.

For Zeta potential measurements, a Universal Dip Cell (ZEN1002) was imersed into sample after size measurement. The Zeta potential was determined by electrophoretic light scattering (ELS) under automatic voltage selection with 300 s delay between measurements using the Smoluchowski model. Individual records represents the average of 15 measurements and for each sample three such records were acquired. The results (expressed in mV) are presented as the averages of these measurements, together with the standard deviation. The results were analyzed using the build-in Zetasizer Software 7.12 (Malvern Instruments).

The stability of nanoemulsions stored at 4 °C for 3 months or at 37 °C for 1 week was checked by periodical measurement of size and zeta potential.

#### 2.4.2. Determination of the Amount of Peptide Coupled to LN Surface

The amount of peptide coupled to the surface of LNs was determined indirectly by measuring the amount of peptide remained uncoupled. The uncoupled peptide was separated by centrifugation of LN samples using Amicon centrifugal filter units of 100 kDa cut-off (Millipore) and was measured in the filtrate. The amount of coupled peptide was then calculated as the difference between the initial amount of peptide introduced in the reaction and the uncoupled one. The concentration of peptide was determined by ultrahigh performance liquid chromatography (UHPLC), as previously described [21] on a UHPLC Agilent Technologies 1290 Infinity instrument equipped with a binary pump, vacuum degasser, column oven, temperature-controlled autosampler and diode array detector (DAD). The chromatographic column was an Eclipse Plus ZORBAX C18 column narrow bore RR (150 mm × 2.1 mm, 3.5 µm), maintained at 25 °C. The mobile phase consisted of 0.1% trifluoroacetic acid in water (solvent A) and 0.1% trifluoroacetic acid in acetonitrile (solvent B). Separation was performed by gradient elution as follows: 0–0.8 min, 5% B; 0.8–8 min, 5–26% B; 8–8.8 min, 26% B; 8.8–10 min, 26–5% B, 10–12 min, 5% B. The flow rate was set at 0.25 mL/min. The injection volume of the test sample was 5 µL and the detection wavelength was 220 nm. Data acquisition and processing were performed using Agilent ChemStation software (B.04.02 Version, Agilent Technologies, Santa Clara, CA, USA).

#### 2.4.3. Flavonoid Entrapment into Lipid Nanoemulsions

To measure the encapsulation efficiency (EE) of flavonoids into LNs, the non-entrapped, free flavonoid was separated from nanoemulsions by centrifugation using Amicon Ultra 100 KDa membrane filter. The concentration of flavonoid in the filtrate and concentrate was measured on a UHPLC Agilent Technologies 1290 Infinity instrument using an Eclipse Plus ZORBAX C18 column narrow bore RR (150 mm × 2.1 mm, 3.5 µm), maintained at 30 °C. The mobile phase consisted of water (solvent A) and acetonitrile (solvent B). Separation was performed by gradient elution as follows: 0–1 min, 23% B; 1–9 min, 23–60% B; 9–10 min, 60% B; 10–12 min, 60–23% B; 12–14 min, 23% B. The flow rate was set at 0.25 mL/min. The injection volume of the test sample was 5 µL and the detection wavelength was 290 nm. Data acquisition and processing were performed using Agilent ChemStation software (B.04.02 Version, Agilent Technologies, Santa Clara, CA, USA).

The concentration of flavonoid in the filtrate and concentrate was measured by HPLC, and EE was calculated utilizing the formula: EE (%) = (Total Flavonoid − Free Flavonoid)/Total Flavonoid.

#### 2.4.4. In Vitro Release of Flavonoid Incorporated in Nanoemulsions

In order to evaluate the capacity of the entrapped flavonoid to be released from the lipid nanoemulsions, we employed the dialysis bag method. For this, equal amonts (340 µg) of flavonoid, either free or incorporated in nanoemulsions, were diluted in phosphate buffer saline (PBS), pH 7.4, to a final volume of 2 mL and dialyzed against a 10-fold excess volume of prewarmed PBS at 37 °C, with shaking at 150 rpm. Aliquots of 100 µL were harvested at indicated time points and assessed for flavonoid content by UHPLC. Volumes of fresh dissolution medium equal to the volumes withdrawn were added to mantain the initial volume and sink conditions.

### 2.5. Cell Culture

To investigate the in vitro effects of flavonoid-loaded lipid nanoemulsions, the human endothelial cell line EA.hy926 (purchased from American Type Culture Collection, Manassas, VA, USA) was employed. Cells were grown in DMEM (Dulbecco’s modified Eagle’s medium) with 1% glucose and supplemented with 10% fetal bovine serum (FBS), 100 U/mL penicillin, 100 μg/mL streptomycin and 50 μg/mL neomycin, at 37 °C in a 5% CO_2_ incubator.

THP-1 human monocytes (American Type Culture Collection) were maintained in RPMI medium supplemented with 10% heat-inactivated FBS, at 37 °C in an incubator supplied with 5% CO_2_.

### 2.6. Viability Assay

EA.hy926 cells were seeded in 96 well plates at a density of 10,000 cells/well. After 24 h, the medium was replaced with fresh serum free medium containing various LN concentrations and incubated for another 24 h. At the end of the period, the medium was replaced with Phenol Red-free medium containing XTT and the intermediate electron transporter phenazine methosulfate (PMS), according to manufacturer’s instructions. After 2 h-incubation at 37 °C, the formazan produced upon the reduction of the tetrazolium salt by the mitochondrial enzymes in the metabolically active cells was measured at 450 nm using a plate reader (TECAN Infinite M200Pro, Tecan Group Ltd., Männedorf, Switzerland). Viability was normalized to the control cells cultured in the absence of any treatment and the results were expressed as percentages (%) of the control considered as 100%.

### 2.7. Uptake of Lipid Nanoemulsions by Endothelial Cells

EA.hy926 cells were seeded in 24-well plates, at a density of 100,000 cells/well and 24 h later were activated with 20 ng/mL TNF-α to induce VCAM-1 expression. After 18 h, the medium was replaced with fresh serum-free medium containing Rhodamine-PE labeled LNs, either non-targeted or VCAM-1 targeted, and cells were incubated for 4 or 24 h. Two different doses of LNs were used, 0.1 and 1.0 µmol lipid/10^6^ cells, respectively. Next, the cellular uptake of LNs was assessed qualitatively by fluorescence microscopy, and quantitatively by flow cytometry. Microscopy investigations were performed on an inverted Olympus IX81 microscope equipped with 40× objective. Micrographs were acquired in the same exposure conditions and processed using CellSens software. To quantify the uptake of Rhodamine-PE labelled LNs, EA.hy926 cells were detached from the wells with trypsin, resuspended in PBS containing 0.5% paraformaldehyde and then analyzed using Gallios Flow Cytometer (Beckman Coulter, Brea, CA, USA) in the FL2 channel. Data analysis was performed with Flowing Software freeware (University of Turku, Turku, Finland).

### 2.8. Monocyte Adhesion Assay

EA.hy926 cells were seeded in 24-well plates at a density of 100,000 cells/well, grown to confluency and treated with 20 ng/mL TNF-α for 18 h. Then, after media removal, the endothelial cells were incubated for 24 h with non-targeted or VCAM-1 targeted LNs encapsulating flavonoids or with free flavonoids at the same concentration (50 μM). Next, the media containing LNs were removed and 250,000 fluorescently labelled THP-1 cells were added per well. THP-1 monocytes were fluorescently labelled by incubation with 1 µg BCECF-AM per 10^6^ cells, for 30 min at 37 °C in RPMI containing 0.05% FBS under gentle shaking. After 30 min incubation at 37 °C, non-adherent THP-1 monocytes were removed by washing with warm RPMI 1640 medium and the cells were analyzed using an Olympus IX81 inverted microscope with the 40× objective using the FITC filter. Afterwards, the cells were lysed with 2% Triton X-100 in 0.1 M NaOH and the fluorescence was measured using a TECAN spectrofluorometer with excitation and emission wavelengths at 485 nm and 535 nm, respectively.

### 2.9. Monocyte Transmigration Assay

Transmigration assays were performed in 24-well plates, equipped with 8.0 µm inserts treated with 0.1% gelatin. EA.hy926 cells were seeded at a density of 10,000 cells/insert, grown to confluency and incubated for 24 h with 20 ng/mL TNF-α in the presence of 50 μM flavonoid either free or loaded into non-targeted or VCAM-1 targeted nanoemulsions. After 24 h, the media from both chambers were removed and 100,000 THP-1 monocytes, fluorescently labeled with BCECF-AM, were added in the upper chamber of each well. To ensure transmigration of monocytes, a FBS gradient was used to mimic chemotactic conditions (1% in the upper chamber and 10% in the lower chamber), as previously reported [19,21,22]. After 18 h, the migrated monocytes in the lower chamber as well as attached to the filter were collected, washed with PBS and the fluorescence was measured using a TECAN spectrofluorometer [19]. The conversion to the monocyte number was performed based on a calibration curve of the fluorescence intensity as a function of the number of fluorescently labeled-monocytes. For this, the fluorescence of a sample consisting of a known number of labelled monocytes and of serial dilutions of this was measured on TECAN spectrofluorometer.

### 2.10. Immunoblotting Assay

To assess the protein expression of pro-inflammatory markers, EA.hy926 cells were seeded in 6 well plates at a density of 250,000 cells/ well, grown to confluency and incubated with 20 ng/mL TNF-α for 18 h. Afterwards, the medium was replaced with fresh serum free medium in which flavonoid-loaded LNs or free compounds were added to a final concentration of 50 μM. After a 24 h incubation at 37 °C, the cells were rinsed with PBS, harvested into Sx2 Laemmli buffer or subjected to preparation of nuclear extracts as described in [24] and protein concentration was measured by Amido Black assay [25]. SDS-PAGE was performed by loading 30 µg protein per lane, followed by transfer onto nitrocellulose membrane and subsequent probing with the appropriate antibody. The following primary antibodies were employed to detect proteins of interest: anti MCP-1 (Abcam. cat. no 9669), anti β-actin (BIO-RAD cat. no. MCA5775GA), anti p65 (Santa Cruz Biotechnology, cat. no.sc-109), anti TBP (Santa Cruz, cat. no. sc421).

### 2.11. Quantitative RT-PCR

RNA isolation and purification were achieved by standard Trizol method, and mRNA was reverse transcribed using the High Capacity cDNA Reverse Transcription Kit following manufacturer’s instructions (ThermoFisher Scientific). SYBR Green-based real-time PCR experiments were performed in triplicates in a 96-well plate on a ViiA7 instrument (Applied Biosystems, Foster City, CA, USA). MCP-1 expression was normalized to β-actin expression and fold changes were calculated by the 2^−ΔΔCT^ method [26]. The primers used were: for human MCP-1 (Ref_Seq NM_002982.3), forward: CAGCCAGATGCAATCAATGCC, and reverse: TGGAATCCTGAACCCACTTCT, resulting in a 190 bp amplicon, and for human ACTB (Ref_Seq NM_001101.4) forward: GACGAGGCCCAGAGCAAGAGAGG, and reverse: CATGGCTGGGGTGTTGAAGGTCTC with a 231 bp corresponding amplicon.

### 2.12. Hemocompatibility Test

Hemolysis assay was performed according to a protocol previously described [27]. The erythrocytes were isolated from blood drawn from C57BL6 or apoE-deficient mice by centrifugation and resuspended at 1:10 ratio in PBS containing 50 µM flavonoids, either free or loaded into non-targeted and VCAM-1 targeted LNs. After one-hour incubation on a rotary shaker at 37 °C, the suspensions were centrifuged to separate erythrocytes, and the hemoglobin released in the supernatants was measured at 540 nm with a TECAN Infinite M200Pro plate reader. Negative control received no treatment, and positive control was obtained by complete erythrocyte lysis in 0.5% Triton X-100. The percentage of lysed cells was calculated as 100× (absorbance of sample-absorbance of negative control)/(absorbance of positive control-absorbance of negative control). Protocols for manipulating animals were approved by the Animal Ethics Committee of our Institute.

### 2.13. Statistical Analysis

The data were analyzed with GraphPad PRISM 7 (GraphPad Software, Version 7, San Diego, CA, USA). Results were expressed as means ± standard deviation. Statistical significance was calculated using a one-tailed *t*-test in the case of adhesion, transmigration and gene expression experiments and double-tailed *t*-test, in the case of cytotoxicity assay. A *p* value less than 0.05 was considered statistically significant. To analyze kinetics of polyphenol release, the data were fitted by one-phase exponential nonlinear regression.

## 3. Results

### 3.1. Preparation and Physicochemical Characterization of Flavonoid-Loaded Nanoemulsions

#### 3.1.1. Size and Zeta Potential

**Figure 1:**
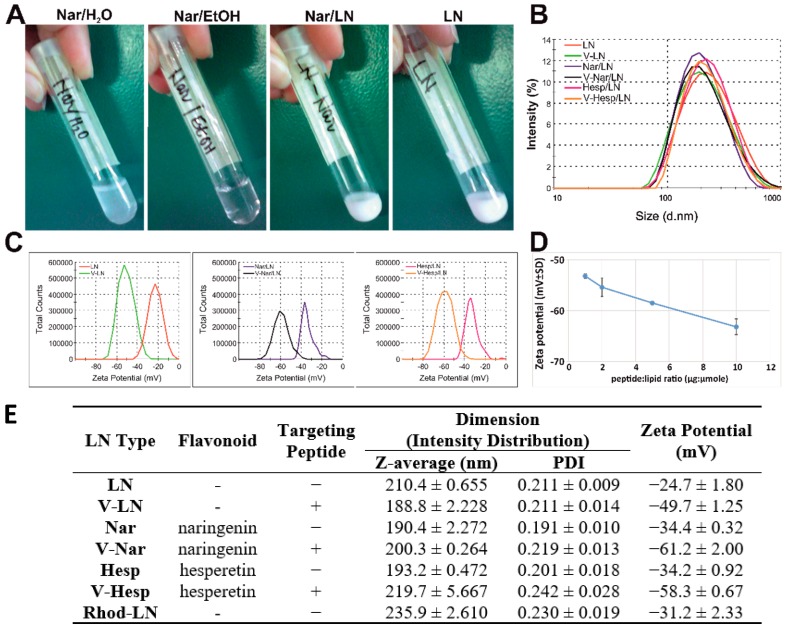
Physico-chemical characterization of flavonoid-loaded lipid nanoemulsions. (**A**) Photographs depicting naringenin dispersed in water (Nar/H_2_O), dissolved in ethanol (Nar/EtOH) or encapsulated into lipid nanoemulsions (Nar/LNs). Empty lipid nanoemulsions are also shown (LNs). (**B**) Particle size distribution by intensity for prepared nanoemulsions as a result of dynamic light scattering (DLS) measurements. (**C**) Zeta potential distribution by electrophoretic light scattering (ELS) measurements for non-targeted and Vascular Cell Adhesion Protein 1 (VCAM-1) targeted nanoemulsions either empty (LNs and V-LNs) or loaded with naringenin (Nar/LNs and V-Nar/LNs) or hesperetin (Hesp/LNs and V-Hesp/LNs). (**D**) Increased peptide: lipid coupling ratio proportionally shifts zeta potential to more negative values. (**E**) Average hydrodynamic diameter and zeta potential of empty and flavonoid-loaded nanoparticles (non-targeted and VCAM-1 targeted). Data are means ± standard deviation (S.D.) from three independent nanoemulsions samples with triplicate measurements each. Coupling ratio in (**B**,**C**,**E**) is 1 µg peptide:1 µmol total lipid. The last row in the table indicates the parameters of Rhodamine-labelled nanoemulsions used in uptake experiments.

To increase the water solubility of the two flavonoids, we incorporated naringenin and hesperetin in a suitable hydrophobic environment provided by lipid nanoemulsions. As a result, while at a concentration of ~3 mM (0.8 mg/mL), naringenin is not soluble in water (Figure 1A, panel I), but in ethanol (Figure 1A, panel II), it was homogeneously incorporated into lipid nanoemulsions (Figure 1A, panel III). The lipid nanoemulsions had a white milky appearance, similar to that of the empty nanoemulsions (Figure 1A, panel IV). The nanoemulsions incorporating hesperetin had the same appearance (not shown). Dynamic light scattering measurements indicated an average hydrodynamic diameter of ~ 200 nm (Figure 1B). Also, the nanoparticle population was homogenous, as reflected by the polydispersity index (PDI) of ~0.2 (Figure 1E). We generated six different types of lipid nanoemulsions, based on the cargo and the functionalization with the VCAM-1 recognition peptide. Size analysis revealed that, independent of the functionalization with the peptide or flavonoid incorporation, there was no significant size difference between the six distinct types of nanoemulsions (Figure 1B,E). Regarding the zeta potential, this was negative in all the cases, and the nanoemulsions were stable with an absolute value larger than the stability threshold of 30 mV [28] with the exception of the empty, non-functionalized LNs which had a slightly smaller value (absolute value around 25 mV) (Figure 1B). Functionalization with peptide increased stability, zeta potential being shifted to negative values of around −50 to −60 mV for a coupling ratio of 1 µg peptide:1 μmol total lipid (Figure 1C,E). A series of coupling ratios of 1, 2, 5, and 10 µg peptide to total mole lipid, equivalent to molar ratios of peptide to Mal-PEG-DSPE of 3.35%, 6.7%, 16.75%, and 33.5% was tested and the shift of zeta potential to more negative values was proportional to the amount of peptide coupled on the surface (Figure 1D). This is in accordance with the fact that VCAM-1 recognizing peptide has an isoelectric point of 8.83 (calculated based on [29]), thus it is positively charged at pH lower than this value (including physiological value), and attracts supplementary negative charges that contribute to the zeta potential.

To verify the stability of the nanoemulsions, we have periodically assessed the size and zeta potential of nanoemulsions, stored at 4 °C for different time intervals up to 3 months or at 37 °C up to 1 week. Representative results of one set of measurements performed on Nar/LNs and V-Nar/LNs are summarized in Table 2. Since, there were no significant deviations of the dimensions and surface charge of the nanoparticles during 3-months storage period, except of slight increase in the PDI at the end of the period, we may safely assume that the prepared LNs can be used within 3-months without major alterations of their properties.

#### 3.1.2. Flavonoid-Encapsulation Efficiency

We aimed to incorporate flavonoids into nanoemulsions at a final concentration of 1 mg/mL, a concentration that exceeds the reported water solubilities at least 4 times. By the aforementioned method we were able to achieve encapsulation efficiencies (expressed as % from total compound we started with) as follows: 86 ± 3 (for Nar/LNs), 79 ± 1 (for V-Nar/LNs), 96 ± 5 (for Hesp/LNs), 81 ± 2 (for V-Hesp/LNs).

#### 3.1.3. In Vitro Flavonoid Release

**Figure 2:**
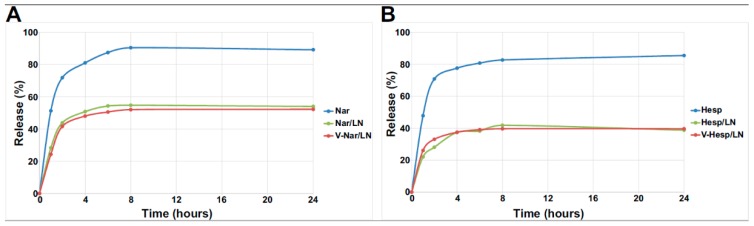
In vitro cumulative release (%) of naringenin (**A**) and hesperetin (**B**) from non-targeted and VCAM-1 targeted LNs or free flavonoids in Phosphate Buffered Saline (PBS), pH 7.4 at 37 °C.

To measure the in vitro release of the compound entrapped in the LNs, we performed studies using the dialysis bag method which showed that flavonoids entrapped into LNs were released slower and to a lower extent than the free compounds starting from the same initial concentration (Figure 2A,B). Data were best fitted by a Weibull model, equivalent to a one phase exponential nonlinear regression y(t) = y0 × (1 − exp(−kt)), where t is the time, y0 is the plateau, k is a constant. The parameters of the fit are summarized in Table 3.

The cumulative release for the free flavonoids approached the expected equilibrium values of ~90% (the remainder up to 100% being retained in the dialysis bag) after 8 h, while in the case of the nanoemulsions, the compounds were released in proportion of only ~50% in the case of entrapped naringenin (Figure 2A) and ~40% in the case of entrapped hesperetin (Figure 2B) at the same time point. In all cases, an initial burst release occurred for both free and entrapped compounds in the two hours, showing a release of approximately 70% (free Nar and Hesp) and ~40%% (Nar/LNs and V-Nar/LNs) and ~30% (Hesp/LNs and V-Hesp/LNs), respectively followed by a relatively modest supplementary release to reach the corresponding plateau. One can notice that the release of flavonoid is independent of the functionalization of the LNs with VCAM-1 recognition peptide, leading to overlapping release profiles for the targeted and non-targeted LNs loaded with the same cargo (Figure 2A, Nar/LNs vs. V-Nar/LNs and Figure 2B, Hesp/LNs vs. V-Hesp/LNs). These kinetic release studies demonstrated a stable encapsulation of polyphenols into nanoemulsions and their prolonged release. Hence, the release kinetics together with the other physico-chemical characteristics recommend the polyphenol-loaded LNs as appropriate nanoformulations for further in vitro and in vivo analysis.

#### 3.1.4. In Vitro Cytotoxicity of Flavonoid-Loaded LNs Assayed on EA.hy926 Cells

**Figure 3:**
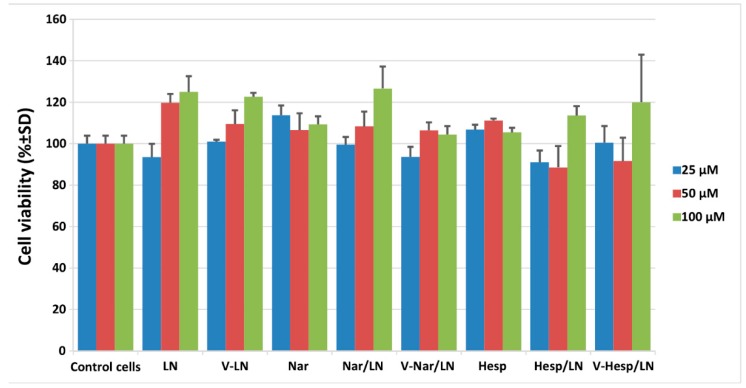
Viability of EA.hy926 cells exposed for 24 h to different concentrations of flavonoid-loaded lipid nanoemulsions or to corresponding concentration of free flavonoids, assessed by XTT assay. Results are presented as means ± standard deviation (S.D.) of a representative experiment performed in triplicates.

As a first step in characterizing the in vitro behavior of flavonoid-loaded nanoemulsions, we performed XTT viability assay using the human endothelial EA.hy926 cells. Our data indicated that upon incubation for 24 h with three increasing doses of nanoemulsions, corresponding to flavonoid concentrations of 25, 50 and 100 µM, there was no significant effect on cell viability (Figure 3). To establish whether the detected variations were significant, data obtained when cells were exposed to different concentrations of flavonoid-loaded lipid nanoemulsions or to corresponding concentration of free flavonoids were compared with the control values, represented by the cells which received no treatment. In none of the cases, were p values less than 0.05. The tested concentrations were proved to be effective in other in vitro studies [30], yet they are much higher than the plasma concentrations following oral ingestion of dietary flavonoids, which do not exceed 10 µM [9,10]. We designed the experiment using these increased concentrations based on the fact that, when a targeted nanoparticle is utilized, a local concentration higher that the average plasma concentration is expected to be reached. Based on the lack of cytotoxicity, as demonstrated by the XTT assay, for the next experiments we chose to use the intermediate tested concentration of 50 µM, as this appears a reasonable local concentration to be reached upon delivery of vectorized nanoparticles.

### 3.2. VCAM-1 Targeted LNs Are Taken up at a Higher Extent by TNF-α Activated EA.hy926 Cells as Compared with Non-Activated Cells

**Figure 4:**
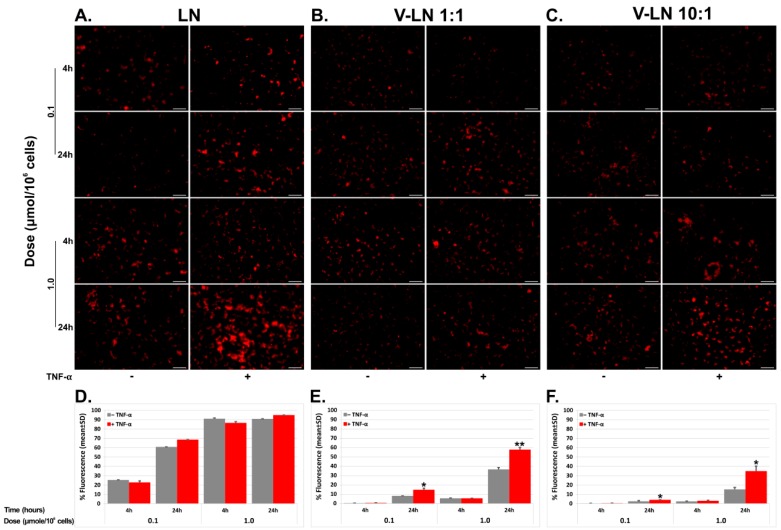
Uptake of Rhodamine-labelled non-targeted (**A**,**D**) or VCAM-1 targeted (**B**,**C**,**E**,**F**) nanoparticles by EA.hy926 cells. Confluent endothelial cells EA.hy926 activated or not with Tumor Necrosis Factor (TNF)-α (20 ng/mL) for 18 h were incubated for the indicated times with two different doses of nanoparticles. Fluorescence micrographs are shown in (**A**–**C**) (scale bar: 50 µm). In (**D**–**F**) a quantitative assessment of the uptake by flow cytometry corresponding to the upper panels (**A**–**C**, respectively). It can be observed that nanoparticles were taken up in a dose- and time-dependent manner and that TNF-α influenced only the uptake of VCAM-1 targeted particles. Coupling ratio of peptide (µg) and total lipid (µmol) was 1:1 (**B**,**E**) and 10:1 (**C**,**F**).

In order to follow the uptake of the flavonoid-loaded LNs, we employed EA.hy926 cells which were exposed to TNF-α to induce the expression of VCAM-1 (as revealed by Western blot, data not shown), and were subsequently incubated for 4 and 24 h with Rhodamine-PE labelled non-targeted (LNs) or VCAM-1 targeted (V-LNs) nanoparticles in two different doses of 0.1 and 1.0 µmol lipid/10^6^ cells. We also employed two different coupling ratios between peptide (µg) and lipid (µmol) of 1:1 (V-LN 1:1) and 10:1 (V-LN 10:1). At the end of the incubation time, we evaluated the uptake of nanoparticles qualitatively by fluorescence microscopy and quantitatively by flow cytometry. We found that both non-targeted and VCAM-1 targeted LNs were taken up by the cells in a dose- and time- dependent manner. However, while the uptake of non-targeted LNs was independent of TNF-α activation, driven by a non-specific endocytic mechanism, the uptake of targeted LNs was receptor-mediated, via VCAM-1 cell adhesion molecule expressed on the endothelial cell surface and thus dependent on TNF-α activation. Also, the uptake of non-targeted LNs reached higher values (higher than 60% at 4 h, Figure 4D) as compared with the V-LNs (for which it reached less than 20% in case of V-LN 1:1 and even less that 5% for V-LN 10:1). At 24 h, the uptake of non-targeted LNs had already reached saturation, while the uptake of targeted nanoparticles was still ongoing, reaching ~60% for V-LN 1:1 and ~30% for V-LN 10:1. There was a ~1.5- and ~2-fold enhancement, respectively, of the uptake of V-LNs upon exposure to TNF-α, as compared with the uptake in the absence of TNF-α for the two coupling ratios analyzed (Figure 4E,F, columns corresponding for the dose of 1.0 µmol lipid and time 24 h).

In the uptake experiments, we found out that a higher coupling ratio of 10:1 (Figure 4, panels C and F) led to a decreased uptake as compared to the 1:1 coupling ratio (Figure 4, panels B and E), a fact that may be due to a steric hindrance at the recognition sites when nanoparticle surfacing is denser. It is recognized that while PEGylation of nanoparticles is a good strategy to lengthen their blood circulation time, it also restrains the interaction of nanoparticles with their neighboring molecules, either other nanoparticles or blood components [31]. Based on this finding, we performed the subsequent experiments at a 1:1 (μg peptide: μmol lipid) coupling ratio.

### 3.3. Flavonoid-Loaded LNs Have a Functional Role in Inhibiting Monocyte Adhesion and Transmigration to/through Activated EA.hy926 Cells

**Figure 5:**
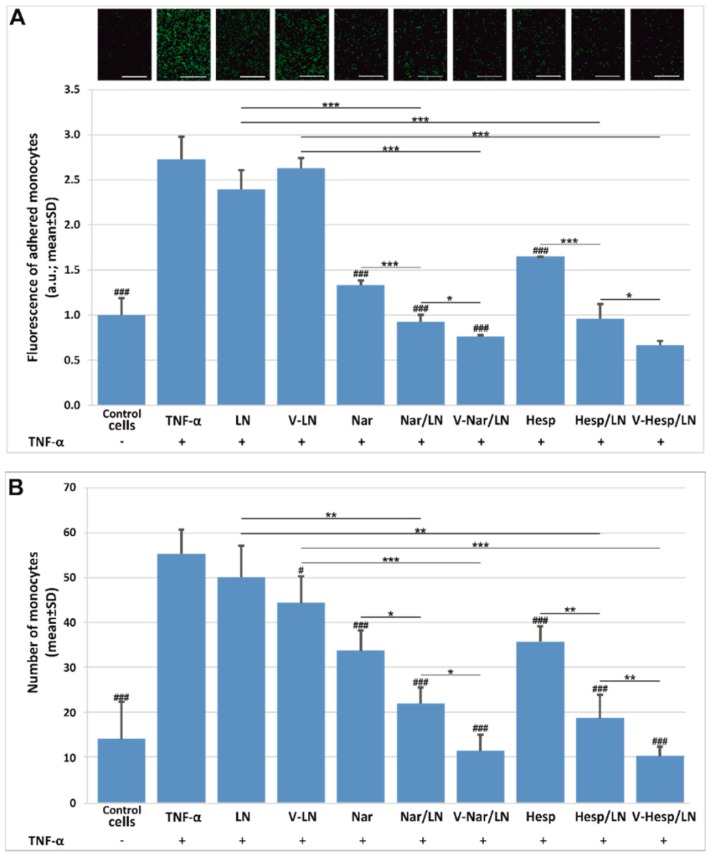
Functional role of VCAM-1 targeted flavonoid-loaded nanoemulsions in the reduction of monocyte adhesion (**A**) and transmigration (**B**) to/through TNF-α activated EA.hy926 cells, as compared with the same concentration of free flavonoids (50 µM). Results are presented as means ± standard deviation (S.D.) of a representative experiment from three experiments performed in triplicates; * depicts comparisons between the groups; # are significances relative to TNF-α, * and # represent *p* < 0.05; ** *p* < 0.01; *** and ### *p* < 0.001.

To validate the anti-inflammatory potential of the flavonoid-loaded LNs we employed the monocyte adhesion and transmigration assays [19,32]. We found that at 50 µM incorporated flavonoid, nanoemulsions reduced both the adhesion (Figure 5A) and transmigration (Figure 5B), to a greater extent than the corresponding free compounds. Also, there was a small, yet significant, supplementary reduction by the VCAM-1 targeted LNs as compared with the non-targeted LNs in both assays. In the case of the transmigration assay (Figure 5B), the empty, VCAM-1 targeted LNs (V-LNs) also exerted a small inhibition in the monocyte movement across the insert (*p* < 0.05), however this was less significant as compared to the effect of the flavonoid-loaded nanoparticles (V-Nar/LNs, respectively V-Hesp/LNs) (*p* < 0.005).

### 3.4. Flavonoid-Loaded LNs Reduce the Expression Level of Pro-Inflammatory Molecules in TNF-α Activated EA.hy926 Cells

**Figure 6:**
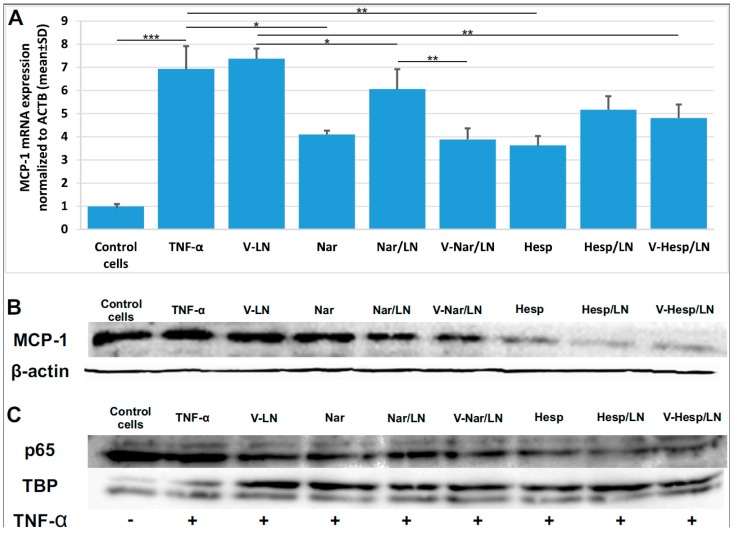
Naringenin (Nar) and hesperetin (Hesp), incorporated into lipid nanoemulsions, decrease pro-inflammatory markers in TNF-α activated EA.hy926 cells. (**A**) Quantitative RT-PCR of MCP-1 mRNA expression in TNF-α activated EA.hy926 cells treated with V-Nar/LNs and V-Hesp-LNs as compared with Nar/LNs, Hesp/LNs and free Nar and Hesp; (**B**) immunoblotting experiments of total cell lysates of TNF-α activated EA.hy926 cells treated as above and probed with anti-MCP1 antibody; (**C**) immunoblotting of nuclear extracts probed for the p65 subunit, relative to the expression of TATA-binding protein (TBP). *, **, *** depict comparisons between the groups; * represent *p* < 0.05; ** *p* < 0.01; *** *p* < 0.001.

To evaluate the expression of prominent pro-inflammatory molecules on EA.hy926 cells exposed to TNF-α and subsequently treated with flavonoid-loaded LNs, we followed two lines of investigation. First, we performed real time PCR and immunoblotting experiments for monocyte chemotactic protein 1 (MCP-1). We found that MCP-1 expression, at both mRNA (Figure 6A) and protein (Figure 6B) levels, is reduced upon incubation with either free or LN-encapsulated flavonoids. Thus, the treatment of the cells with Nar/V-LN and Nar/LN determined a reduction of ~50% and ~25%, respectively, in MCP-1 mRNA expression as compared with levels obtained in non-treated, TNF-α activated cells. Consequently, a statistically significant difference between the non-targeted and targeted LNs occurred only for naringenin, while in case of hesperetin both types of LNs displayed similar efficacies. Real time PCR results are further supported by the data obtained at protein level, which proved the anti-inflammatory potential of flavonoid-loaded LNs through the reduction of MCP-1. Secondly, we bring evidence that treatment of the TNF-α activated EA.hy926 cells with encapsulated flavonoids consistently reduced the nuclear translocation of the dimeric transcription factor NF-κB (Figure 6C), as probed for the p65 subunit referenced to the nuclear TATA-binding protein (TBP).

### 3.5. Hemocompatibility of Flavonoid-Loaded LNs

**Figure 7:**
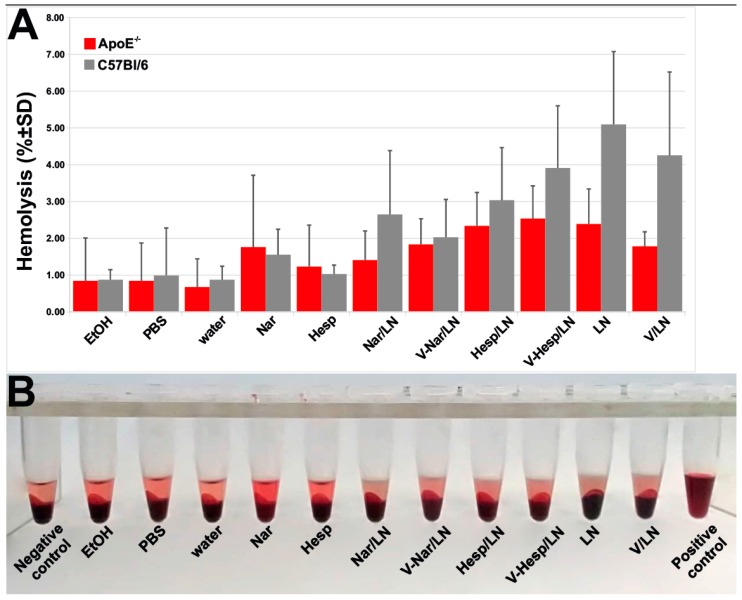
Flavonoid-loaded nanoemulsions did not cause lysis of erythrocytes from apoE-deficient or C57 Black mice. (**A**) Quantification of hemolysis by measuring the absorbance at 540 nm. (**B**) Photographs of the pelleted erythrocytes upon exposure to various experimental conditions.

Since we intend to further investigate the therapeutic potential of VCAM-1 targeted LNs in preclinical animal models after intravenous administration, we evaluated their hemolytic activity, as this is one important test when assaying biomaterials hemocompatibility [33]. We investigated the impact of flavonoid-loaded LNs on erythrocytes integrity isolated from two murine models-C57BL6 and apoE-deficient mice. We found that the percentages of lysed erythrocytes after incubation with flavonoid-loaded nanoemulsions did not exceed the 5% threshold (Figure 7), considered to be the critical safe hemolytic ratio for biomaterials, according to International Organization for Standardization (ISO) 10993-4:2017.

## 4. Discussion

The pharmacological potential of the main citrus flavonoids, naringenin and hesperetin, is underlined by the increasing number of papers which evidence their benefits in a variety of inflammation-related diseases [34,35]. As recent research emphasized, various apparently unrelated pathologies, such as cancer, cardiovascular diseases, neurodegeneration, may originate from a common ground, the low grade chronic inflammation “metaflammation” [36]. This unifying concept helps rationalize the extremely diverse actions such as antioxidant, antitumoral, antiadipogenic, hepatoprotective, and cardioprotective effects that were described for citrus flavonoids by focusing them towards one particular aspect: the anti-inflammatory potential. Numerous reports have specifically addressed flavonoid-targeted anti-inflammatory pathways both in vitro and in vivo. The underlying molecular mechanisms relate, but are not restricted, to the reduced expression of adhesion molecules, impaired secretion of cytokines and pro-inflammatory transcription factors (e.g., NF-κB) and upregulation of anti-inflammatory molecules (e.g., transcription factor Nrf2) [37,38,39]. However, despite the multitude of epidemiological and clinical studies and meta-analyses which document the favorable effects of citrus consumption for the prevention and treatment of diverse pathologies, citrus flavonoids are still far from a large-scale therapeutic use due to their poor solubility and bioavailability.

In its normal physiological state, the endothelium presents a non-adherent surface for blood leukocytes but as it becomes progressively activated, there is an increased expression of cell adhesion molecules and chemokines, promoting the recruitment and sub-endothelial transmigration of leukocytes that lead to vascular inflammation [40]. Thus, due to its involvement in the development and progression of vascular inflammation, the endothelium is a good target for therapeutic intervention. We have previously demonstrated that VCAM-1, a cell adhesion molecule which is specifically expressed on the cytokine-activated endothelium, is an appropriate target for directing drugs encapsulated into nanoparticles to activated ECs [21,22,41]. In the current work, we developed nanoformulations of flavonoids directed towards VCAM-1 expressed on the surface of the activated endothelium with the purpose to reduce the EC inflammation. To this aim, we coupled a VCAM-1 recognition peptide to the surface of flavonoid-loaded lipid nanoemulsions. The obtained nanoemulsions showed a hydrodynamic size of ~200 nm and incorporated flavonoids at a concentration of up to at least four-fold their water solubility. Moreover, by measuring periodically size and zeta potential of nanoemulsions stored at 4 °C for 6 weeks or at 37 °C for 1 week we did not notice significant changes in these parameters. Regarding the in vitro release of their flavonoid cargo, our data obtained by the dialysis bag method demonstrated a clear-cut difference between the release of free flavonoids and that of the flavonoids entrapped into LNs. While the non-encapsulated compounds rapidly passed through the dialysis membrane, the nanoparticles released the entrapped polyphenols with a slower kinetics. Furthermore, hesperetin, the more hydrophobic of the two compounds is released to a lower extent than naringenin, probably due to a stronger retention within the nonpolar milieu of the nanoparticles. The model that fits best the data was the Weibull model, which points out a one-phase release profile of polyphenols from lipid nanoemulsions [42]. Release from nanoemulsions is a complex process, the encapsulated compound has to pass both through the barrier of nanoemulsions, as well as through the dialysis membrane, therefore there are many variables to be taken into account. In a similar way, the Weibull model was recently shown to describe well the behavior of plumbagin release for nanoemulsions [43]. The slower release from nanoemulsions is important as it may preserve the cargo for longer time as compared with the free compounds which circulate in the blood and are thus susceptible to rapid metabolization.

As for the safety of the prepared LNs, we have showed that in vitro they were nor cytotoxic to EA.hy926 cells by the XTT assay, neither did they provoke lysis of red blood cells from both C57 black 6 and apoE-deficient mice.

The prepared LNs were taken up by the human endothelial cells EA.hy926 in a time- and dose-dependent manner. Nonetheless, a distinct mechanism drives the uptake of VCAM-1 targeted LNs as compared to the non-targeted ones. While the uptake of non-targeted LNs is not influenced by the activation of ECs with TNF-α, the uptake of VCAM-1 targeted LNs is significantly increased ~2-fold in the TNF-α activated ECs as compared with non-activated ECs. Interestingly, the non-specific endocytic mechanisms did not operate for the uptake of VCAM-1 targeted nanoemulsions, even in the non-activated EC, where VCAM-1 expression is low [41]. We consider that the presence of the VCAM-1 recognizing peptide on the surface of LNs may hinder the non-specific uptake of the targeted nanoparticles, leading thus to a lower incorporation by the cells. We assume that this behavior is due to the fact that the targeted nanoparticles possess a higher negative zeta potential (~−50 mV) than the non-targeted ones (~−25 mV). Thus, electrostatic repulsions may be more intense and preclude the uptake of targeted nanoparticles. These interactions may also be important in reducing the uptake of the nanoparticles with more peptide coupled on the surface (V-LN 10:1) as compared with the ones with lower peptide (V-LN 1:1), since there is a further shift in the zeta potential towards more negative values with increased peptide on the surface. A comprehensive study of the mechanisms/pathways involved in the internalization of VCAM-1 targeted and non-targeted LNs requires further investigation. The functional assays of monocyte adhesion (Figure 5A) and transmigration (Figure 5B) to/through ECs proved the efficacy of EC treatment with both VCAM-1 targeted and non-targeted flavonoid-loaded LNs, with a higher efficiency displayed by the flavonoids incorporated into LNs as compared with the free, non-encapsulated ones. A small, yet statistically significant supplementary reduction in monocyte adhesion of about 9.5% for naringenin and 5% for hesperetin-loaded LNs was achieved when using VCAM-1 targeted nanoparticles, as compared with the non-targeted ones. The effect is even more prominent in case of the transmigration assay that resulted in a reduction of 33% and 25% in the transmigration of monocytes through EC treated with VCAM-1 targeted nanoemulsions-encapsulated naringenin and hesperetin, respectively.

We also brought evidence that underlying molecular mechanisms comprised the decrease of MCP-1 expression and the diminished nuclear translocation of NF-kB in TNF-α activated ECs treated with flavonoid-loaded LNs (Figure 6). Indeed, both MCP-1 [44,45] and NF-κB [46] are two key molecules involved in the activation and dysfunction of endothelial cells and the reduction of MCP-1 expression, respectively of NF-kB nuclear translocation is expected to be beneficial for restoring the normal endothelial status.

Our data align to an emerging trend to incorporate citrus flavonoids into nanocarriers for diverse applications, ranging from topical applications [47] to the improvement of antitumoral action of approved drugs [48]. In the latter case, a notable result was the co-delivery of the anticancer drug tamoxifen and naringenin in a self-nano-emulsifying system in a model of dimethylbenzanthracene-induced breast cancer in female Wistar rats. Naringenin potentiated the anticancer activity of tamoxifen and led to reduced tumor size due to its anti-proliferative effects and P-glycoprotein efflux inhibitory activity. Less attempts were made to specifically deliver citrus flavonoids to pathological sites, thus increasing their local effective concentration. A promising result was the incorporation of naringenin into hyaluronic acid decorated nanoparticles to be efficiently delivered in lung cancer therapy [49].

The data presented herein synergized for the first time the anti-inflammatory therapeutic potential of citrus flavonoids with the directional delivery guided towards cell adhesion molecules, in an attempt that successfully reduced in vitro the effects of two master pro-inflammatory molecules, MCP-1 and the transcription factor NF-κB. Also, they highlight the importance of continuing to devise targeting strategies by appropriate nanoparticle surfacing to exploit the underused pharmacological potential of citrus flavonoids. VCAM-1 targeted flavonoid loaded-LNs developed in this study showed their anti-inflammatory efficacy in vitro and we expect that, in vivo, they will specifically accumulate at the inflamed sites, while the non-targeted LNs will manifest a more even distribution, thus dissipating the efficacious concentration of carried flavonoid. Our and others previous data support the assumption that the presence of PEG_2000_ on the surface of nanoemulsions will assure an increased survival of targeted LNs into blood circulation by reducing the interaction with blood components and the uptake by the mononuclear phagocytic cells.

Further studies will be performed next to evaluate the endothelium targeted delivery and the therapeutic potential of VCAM-1 targeted flavonoid-loaded LNs in vivo, after intravenous administration into animal models of inflammation.

## 5. Conclusions

In this study we successfully incorporated naringenin and hesperetin, two flavonoids with poor hydrosolubility into lipid nanoemulsions, either non-targeted or targeted to the cell adhesion molecule VCAM-1. These nanoemulsions displayed good in vitro stability, and slow release of the cargo. Furthermore they did not exhibit in vitro cytotoxicity as assessed on the endothelial cell line EA.hy926, nor did they provoke lysis of mouse erythrocytes. The flavonoid-loaded LNs exerted anti-inflammatory effects as supported by functional monocyte adhesion and transmigration assays and reduced the expression of the pro-inflammatory molecule MCP-1 and the nuclear translocation of NF-κB.

## Figures and Tables

**Table 1:** Biochemical details of flavonoids incorporated into nanoemulsions (chemical structure, International Union of Pure and Applied Chemistry (IUPAC) name, Chemical Abstracts Service (CAS) identifier (ID), main glycosides and main citrus source).

Flavanone	Naringenin	Hesperetin
Structure	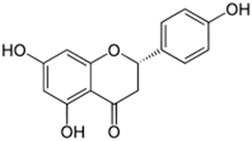	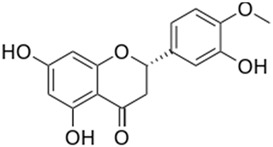
IUPAC Name	5,7-Dihydroxy-2-(4-hydroxyphenyl) chroman-4-one	(*S*)-2,3-Dihydro-5,7-dihydroxy-2-(3-hydroxy-4-methoxyphenyl)-4*H*-1-benzopyran-4-one
CAS ID	480-41-1	520-33-2
Main Glycosides	naringin(naringenin-7-neohesperidoside) narirutin (naringenin-7-rutinoside)	hesperidin (hesperetin 7-rutinoside) neohesperidin (hesperetin 7-neohesperidoside)
Main Citrus Source	grapefruit	orange

**Table 2:** Summary of dimensions and zeta potential for naringenin-loaded non-targeted (Nar/LNs) and targeted (V-Nar/LNs) nanoemulsions, upon storage at 4 °C for different time intervals up to 6 weeks (A) and at 37 °C up to 1 week (B).

**A. Storage at 4 °C**		**Time: 0**	**2 weeks**		**4 weeks**		**6 weeks**	**3 months**
**Nar/LN**	Size (nm)	208.2 ± 1.6	205.6 ± 0.9		203.9 ± 1.1		205.7 ± 0.4	204.3 ± 0.3
	PDI	0.19 ± 0.015	0.181± 0.014		0.181± 0.019		0.186 ± 0.02	0.214 ± 0.006
	Zeta potential (mV)	−35.3 ± 5.1	−35.5 ± 0.3		−32.6 ± 3.1		−33.8 ± 0.5	−38.1± 3.6
**V-Nar/LN**	Size (nm)	210.2 ± 1.6	214.4 ± 2.2		214.3 ± 0.9		217.1 ± 3.2	227.1 ± 3.3
	PDI	0.200 ± 0.01	0.205 ± 0.037		0.183 ± 0.03		0.189 ± 0.02	0.223± 0.012
	Zeta potential (mV)	−52.2 ± 2.3	−53.5 ± 2.06		−55.6 ± 1.7		−54.3 ± 1.4	−55.1 ± 1.1
**B. Storage at 37°C**		**Time: 0**		**1 day**		**3 days**		**7 days**
**Nar/LN**	Size (nm)	206.2 ± 1.8		206.0 ± 1.3		197.9 ± 2.5		202.4 ± 0.2
	PDI	0.217 ± 0.008		0.199 ± 0.01		0.219 ± 0.009		0.174 ± 0.003
	Zeta potential (mV)	−29.1 ± 1.6		−28.7 ± 1.1		−27.6 ± 1.3		−25.6 ± 1.8
**V-Nar/LN**	Size (nm)	214.0 ± 1.3		204.3 ± 1.3		205.8 ± 1.015		206.0 ± 0.7
	PDI	0.224 ± 0.009		0.219 ± 0.009		0.233 ± 0.005		0.22 ± 0.004
	Zeta potential (mV)	−48.7 ± 1.1		−49.5 ± 2.7		−49.1 ± 0.6		−52.0 ± 0.4

**Table 3:** Parameters for polyphenol release data fitted with one phase exponential nonlinear regression.

Parameter	Free Nar	Nar/LNs	V-Nar/LNs	Free Hesp	Hesp/LNs	V-Hesp/LNs
Plateau y0	88.28	54.46	51.98	82.67	39.74	39.08
Half-time	0.8301	0.9046	0.9802	0.7785	0.9957	0.6757
R^2^	0.9962	0.9985	0.9954	0.9959	0.9893	0.9972
Sum of squares	24.54	3.795	10.52	23.18	13.96	3.463
